# Supplementary figures and images for: The tomato yellow leaf curl virus C4 protein alters the expression of plant developmental genes correlating to leaf upward cupping phenotype in tomato
Source: PLoS One. 2022 May 12;17(5):e0257936. doi: 10.1371/journal.pone.0257936 (PMC9098041; doi:10.1371/journal.pone.0257936)

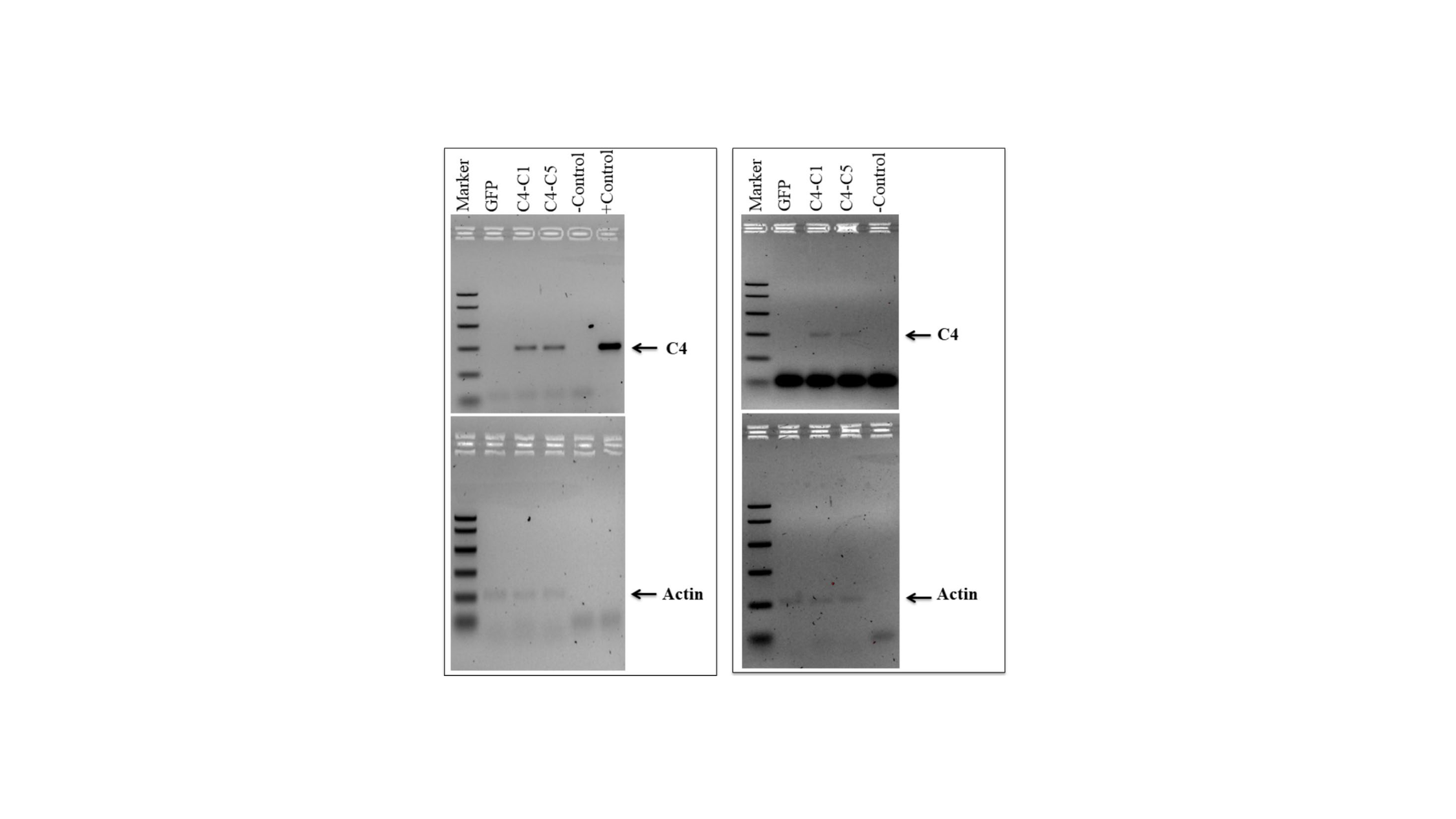

Supplement: S1 Fig — (TIF) [file pone.0257936.s001.tif]

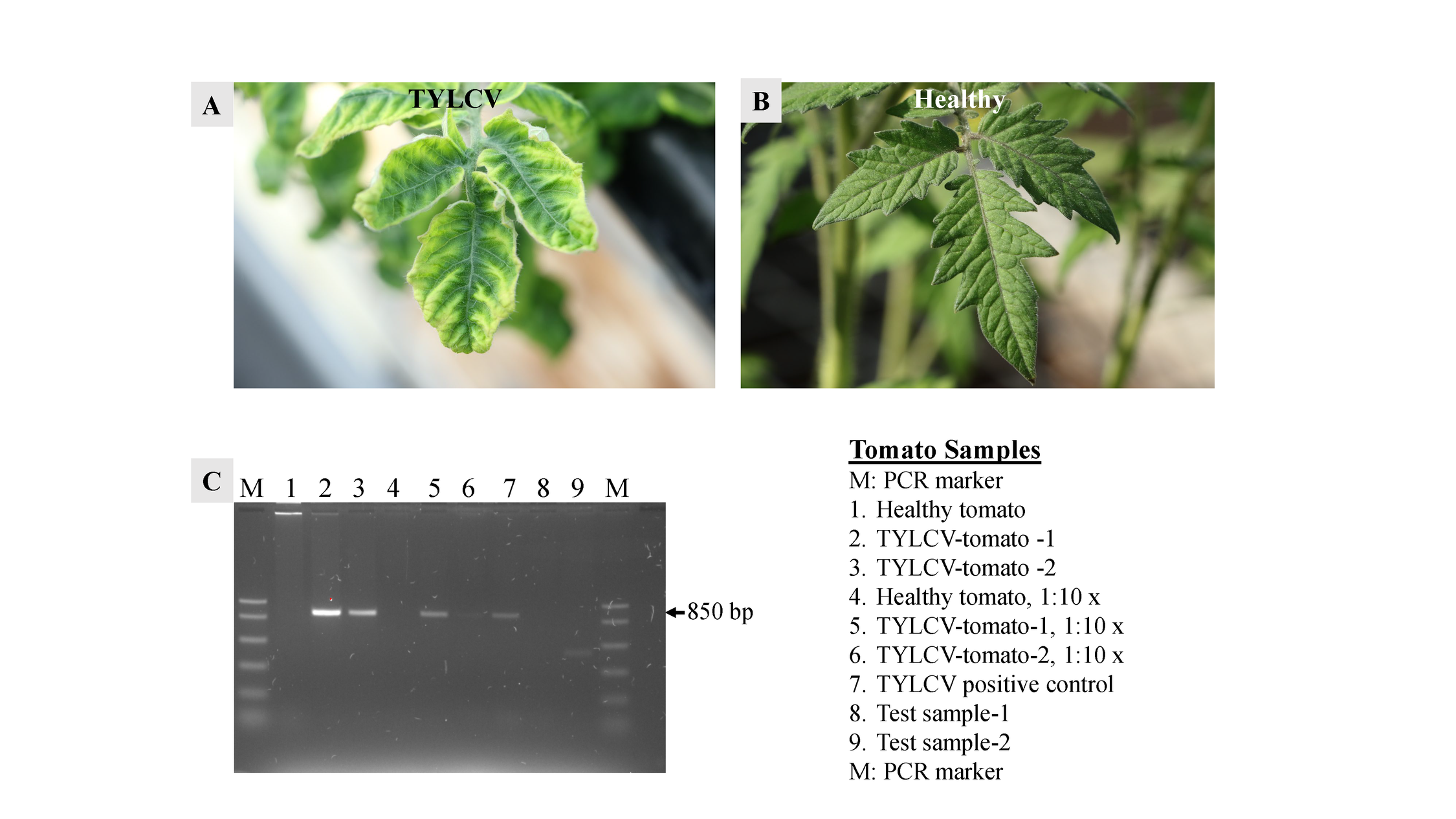

Supplement: S2 Fig — A. TYLCV-infected tomato plant. B. Healthy tomato plant. C. PCR analysis of tomato plants infected with TYLCV. (TIF) [file pone.0257936.s002.tif]
