# Supplementary material for: The tomato yellow leaf curl virus C4 protein alters the expression of plant developmental genes correlating to leaf upward cupping phenotype in tomato
Source: PLoS One. 2022 May 12;17(5):e0257936. doi: 10.1371/journal.pone.0257936 (PMC9098041; doi:10.1371/journal.pone.0257936)
Supplement: S1 Table — (DOCX) [file pone.0257936.s003.docx]

**Supplementary Table S1.** Reads summary for the RNA-Seq libraries.

| **Sample** | **Total raw reads** | **Remove adapter, low quality** | **rRNA reads** | **Poly A reads** | **Cleaned reads** | **Mapped to tomato genome** | |
| --- | --- | --- | --- | --- | --- | --- | --- |
|  |  |  |  |  |  | **No. reads** | **%mapped** |
| C4-C1-1 | 21,105,703 | 17,953,042 | 638,252 | 233,328 | 17,081,462 | 15,516,259 | 90.84 |
| C4-C1-2 | 31,767,336 | 27,327,302 | 570,078 | 96,939 | 26,660,285 | 24,980,499 | 93.70 |
| C4-C1-3 | 20,035,696 | 17,354,700 | 292,674 | 55,935 | 17,006,091 | 15,873,495 | 93.34 |
| GFP1-1 | 14,743,532 | 12,865,416 | 297,553 | 8,861 | 12,559,002 | 11,783,148 | 93.82 |
| GFP1-2 | 20,296,835 | 17,199,746 | 269,728 | 19,898 | 16,910,120 | 15,907,853 | 94.07 |
| GFP1-3 | 14,859,106 | 12,677,950 | 176,573 | 33,932 | 12,467,445 | 11,829,329 | 94.88 |
