# Supplementary material for: The tomato yellow leaf curl virus C4 protein alters the expression of plant developmental genes correlating to leaf upward cupping phenotype in tomato
Source: PLoS One. 2022 May 12;17(5):e0257936. doi: 10.1371/journal.pone.0257936 (PMC9098041; doi:10.1371/journal.pone.0257936)
Supplement: S2 Table — (DOCX) [file pone.0257936.s004.docx]

**Supplementary Table S2.** Pearson correlation coefficient among replicate libraries indicate the reproducibility of RNA-seq libraries.

| **Sample** | **C4-C-1** | **C4-C-2** | **C4-C-3** | **GFP1-1** | **GFP1-2** | **GFP1-3** |
| --- | --- | --- | --- | --- | --- | --- |
| C4-C-1 | 1 | 1 | 1 | 1 | 1.00 | 0.99 |
| C4-C-2 | 1 | 1 | 1 | 1 | 0.99 | 1.00 |
| C4-C-3 | 1 | 1 | 1 | 1 | 0.99 | 1.00 |
| GFP1-1 | 0.99 | 0.99 | 1 | 1 | 0.99 | 0.99 |
| GFP1-2 | 1 | 0.99 | 1 | 1 | 1.00 | 0.99 |
| GFP1-3 | 0.99 | 1 | 1 | 1 | 0.99 | 1.00 |
