# Supplementary material for: The tomato yellow leaf curl virus C4 protein alters the expression of plant developmental genes correlating to leaf upward cupping phenotype in tomato
Source: PLoS One. 2022 May 12;17(5):e0257936. doi: 10.1371/journal.pone.0257936 (PMC9098041; doi:10.1371/journal.pone.0257936)
Supplement: S3 Table — (DOCX) [file pone.0257936.s005.docx]

**Supplementary Table S3.** Validation of selected differentially expressed genes in naturally TYLCV-infected tomato plants using qRT-PCR in comparison with RNA-seq and qRT-PCR in transgenic C4 plants

| Gene ID | Annotation | RNA-seq | qRT-PCR on transgenic C4- plant | qRT-PCR on TYLCV-plant |
| --- | --- | --- | --- | --- |
|  |  | log2fold | log2fold | log2fold |
| Solyc06g054570 | Glutaredoxin | 4.23 | 2.44 | 1.96 |
| Solyc01g096320 | HD-ZIP transcription factor | 3.10 | 0.90 | 0.79 |
| Solyc02g091690 | bHLH transcription factor | -2.40 | -2.30 | -0.67 |
| Solyc05g007230 | Receptor-like kinase, RLK | -2.83 | -1.22 | -2.06 |
| Solyc02g071730 | MADS box transcription factor | 2.59 | 2.79 | 0.39 |
| Solyc03g116890 | WRKY transcription factor | 2.05 | 1.18 | 4.30 |
| Solyc01g067460 | Glutaredoxin | 1.95 | 1.22 | 1.37 |
| Solyc04g011880 | Glutaredoxin | 1.63 | 0.54 | 0.53 |
| Solyc11g073120 | MYB transcription factor | 1.55 | 0.23 | 0.56 |
| Solyc09g015840 | Receptor-like kinase | -1.51 | -1.21 | 1.12 |
| Solyc01g010910 | MYB transcription factor | -1.51 | -2.83 | 2.62 |
